# Hypoxic Tumor Kinase Signaling Mediated by STAT5A in Development of Castration-Resistant Prostate Cancer

**DOI:** 10.1371/journal.pone.0063723

**Published:** 2013-05-10

**Authors:** Kathrine Røe, Åse Bratland, Ljiljana Vlatkovic, Harald Bull Ragnum, Marie Grøn Saelen, Dag Rune Olsen, Laure Marignol, Anne Hansen Ree

**Affiliations:** 1 Department of Oncology, Akershus University Hospital, Lørenskog, Norway; 2 Department of Clinical Molecular Biology and Laboratory Sciences, Akershus University Hospital, Lørenskog, Norway; 3 Department of Radiation Biology, The Norwegian Radium Hospital, Oslo University Hospital, Oslo, Norway; 4 Department of Oncology, The Norwegian Radium Hospital, Oslo University Hospital, Oslo, Norway; 5 Department of Pathology, The Norwegian Radium Hospital, Oslo University Hospital, Oslo, Norway; 6 Department of Tumor Biology, The Norwegian Radium Hospital, Oslo University Hospital, Oslo, Norway; 7 Institute of Clinical Medicine, University of Oslo, Oslo, Norway; 8 Faculty of Mathematics and Natural Sciences, University of Bergen, Bergen, Norway; 9 Prostate Molecular Oncology Research Group, Academic Unit of Clinical and Molecular Oncology, Institute of Molecular Medicine, St. James’s Hospital and Trinity College, Dublin, Ireland; Clermont Université, France

## Abstract

In this study, we hypothesized that androgen-deprivation therapy (ADT) in prostate cancer, although initially efficient, induces changes in the tumor kinome, which subsequently promote development of castration-resistant (CR) disease. Recognizing the correlation between tumor hypoxia and poor prognosis in prostate cancer, we further hypothesized that such changes might be influenced by hypoxia. Microarrays with 144 kinase peptide substrates were applied to analyze CWR22 prostate carcinoma xenograft samples from ADT-naïve, androgen-deprived (AD), long-term AD (ADL), and CR disease stages. The impact of hypoxia was assessed by matching the xenograft kinase activity profiles with those acquired from hypoxic and normoxic prostate carcinoma cell cultures, whereas the clinical relevance was evaluated by analyzing prostatectomy tumor samples from patients with locally advanced disease, either in ADT-naïve or early CR disease stages. By using this novel peptide substrate microarray method we revealed high kinase activity mediated by signal transducer and activator of transcription 5A (STAT5A) in CR prostate cancer. Additionally, we uncovered high STAT5A kinase activity already in regressing ADL xenografts, before renewed CR growth was evidenced. Finally, since increased STAT5A kinase activity also was detected after exposing prostate carcinoma cells to hypoxia, we propose long-term ADT to induce tumor hypoxia and stimulate STAT5A kinase activity, subsequently leading to renewed CR tumor growth. Hence, the study detected STAT5A as a candidate to be further investigated for its potential as marker of advanced prostate cancer and as possible therapeutic target protein.

## Introduction

The initial growth of a malignant prostate tumor is stimulated by androgens [1], and standard first-line treatment of patients with advanced prostate cancer includes androgen-deprivation therapy (ADT) [2]. Although initially effective, tumors inevitably recur in a castration-resistant (CR) state. Metastatic, CR prostate cancer is still remaining the most apprehensive aspect in prostate cancer management, defying prolonged treatment effects. Hence, improved understanding of the driving forces behind CR prostate cancer is warranted to enable development of more efficacious treatment strategies for this group of patients.

Hypoxia is a common microenvironmental factor of solid tumors, promoting tumor growth as well as angiogenesis, metastasis, and therapy resistance [3], [4]. In prostate cancer, tumor hypoxia has indeed been correlated to poor prognosis [5]–[7], although skepticism remains as to its role and routine clinical importance. *In vitro* studies have shown that hypoxia increases the activity and sensitivity of the androgen receptor [8], which in many CR tumors may lead to a selection of adapted phenotypes [9].

It has for many years been well known that disease progression and development of treatment resistance in cancer generally are characterized by altered expression and activity of key mediators in cellular signal transduction, regulating processes such as proliferation, invasion, and angiogenesis [10]. The significance and potential use of such changes to improve cancer treatment are increasingly being recognized, specifically as aberrant protein kinases are playing significant roles in malignant tumor progression [11]–[13]. Of the human kinome (all kinases), approximately half of the tyrosine kinase complement is implicated in cancer [14]. Identification of aberrant tyrosine kinases has therefore emerged as an attractive approach in the quest for improved detection of disease aggressiveness and novel therapeutic targets.

We hypothesized that ADT in prostate cancer, although initially efficient, induces changes in the activity of the tumor kinome that subsequently lead to renewed, CR tumor growth. To examine this hypothesis, microarrays with kinase peptide substrates, mainly representing tyrosine residues, were applied to analyze samples from an *in vivo* preclinical prostate cancer model reflecting different stages in development of CR disease. The impact of hypoxia was assessed by matching this model’s kinase activity profiles with those generated from hypoxic and normoxic prostate carcinoma cell cultures. Clinical relevance was evaluated by analyzing prostatectomy tumor samples from patients with locally advanced disease, either in ADT-naïve or early CR disease stages.

## Materials and Methods

### Ethics Statement

All animal experiments were performed in accordance with protocols approved by the Animal Care and Use Committee at Department of Comparative Medicine, Oslo University Hospital (Permit Number: 32-1984), in compliance with guidelines on animal welfare of the Norwegian National Committee for Animal Experiments. The clinical study protocol was approved by the Regional Committee for Medical and Health Research Ethics (REC South East, Permit Number: S-08607b) and was in accordance with the Helsinki Declaration. Written informed consent was required for participation.

### Preclinical Tumor Model

Tissue fragments (∼2×2×2 mm^3^) of the human, androgen-sensitive CWR22 prostate carcinoma xenograft [15] were subcutaneously (s.c.) implanted into one flank of male, BALB/c nude mice (30–35 g, 6–8 weeks old), together with a 12.5 mg sustained release testosterone pellet (Innovative Research of America, Sarasota, FL).

**Figure 1:**
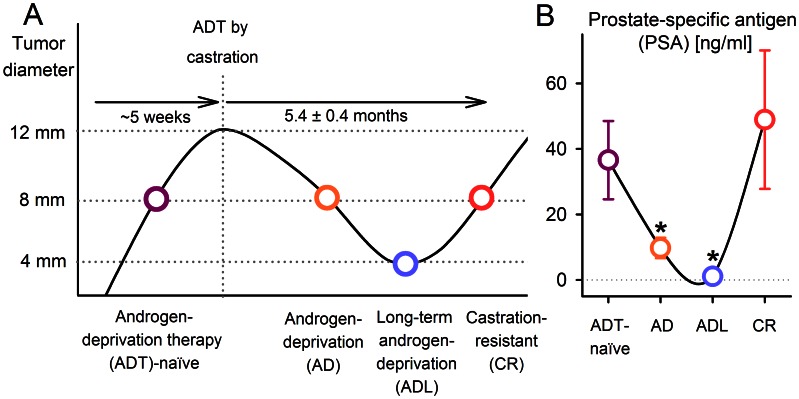
Experimental design. (A) Xenograft diameters versus time in the transition from androgen-deprivation therapy (ADT)-naïve, via androgen-deprived (AD) and long-term AD (ADL), to castration-resistant (CR) disease, after ADT by castration. The time to develop CR disease in patients is ∼2 to 5 years. In our prostate carcinoma xenograft model, the corresponding time was 5.4±0.4 months. The four circles represent the four experimental groups in this study. Blood samples for prostate-specific antigen (PSA) measurements were withdrawn from all of the mice before tumors were excised and snap-frozen for kinase activity profiling. (B) Serum PSA levels reflected the clinical pattern of disease progression. PSA was decreased in AD and ADL tumors, before being restored in CR tumors. Significant differences (p<0.05) compared to ADT-naïve tumors are indicated (*). Each bar represents mean and s.e.m of 3–6 tumors.

The course of development of CR disease following ADT is illustrated in Figure 1A, using the preclinical model to elicit different disease stages. First, xenografts representing ADT-naïve disease were recruited when their shortest diameter reached 8 mm. Second, ADT was performed when the shortest xenograft diameter reached 12 mm, by surgical castration and simultaneous discontinuation of exposure to the testosterone pellet. Androgen-deprived (AD) xenografts were recruited when their diameter was reduced to 8 mm (about 1 month post-castration). Third, long-term AD (ADL) xenografts were recruited when their diameter was reduced to 4 mm (about 4 months post-castration). Last, CR xenografts with renewed growth were included when their diameter reached 8 mm, being 5.4±0.4 months after castration. Xenograft diameters were measured by a caliper twice weekly from implantation until castration and once weekly after castration until the end of the experiment.

Castration was performed under anesthesia, using s.c. injections of zoletil mixture (2.4 mg/ml tiletamine, 2.4 mg/ml zolazepam (Zoletil vet, Virbac Laboratories, Carros, France), 3.8 mg/ml xylazine (Narcoxyl vet, Roche, Basel, Switzerland), and 0.1 mg/ml butorphanol (Torbugesic, Fort Dodge Laboratories, Fort Dodge, IA)), diluted 1∶5 in sterile water, in a dose of 7.5 µl/g. Analgesia was given to castrated animals as s.c. injections of buprenorphine (Temgesic, Schering-Plough, Brussels, Belgium), in a dose of 0.1 mg/kg.

Blood samples for prostate-specific antigen (PSA) analysis were withdrawn from all animals’ femoral veins right before euthanasia. The samples were allowed to coagulate before being centrifuged and stored at −80°C until all samples were analyzed simultaneously. Free and total serum PSA levels were assayed by the fluoroimmunonometric AutoDELFIA ProStatus™ PSA Free/Total kit (PerkinElmer Life and Analytical Sciences, Wallac Oy, Turku, Finland). Euthanasia of animals was performed by cervical dislocations and immediately followed by excision of tumor tissue, which directly was snap-frozen in liquid nitrogen, and stored at −80°C.

### Patients

For the purpose of validating preclinical data, four prostate cancer patients that had undergone radical prostatectomy with clear resection margins were included in the study (Table 1). The patients were recruited at the Norwegian Radium Hospital, Oslo University Hospital. Histopathologic tumor and nodal status (pTN), tumor Gleason score, and preoperative serum PSA levels were recorded as part of standard clinical procedures. Patient 4 had received prolonged (∼2 years) three-monthly s.c. goserelin at a dose of 10.8 mg until his PSA level started to increase following the ADT nadir, and he was referred to definitive surgery.

**Table 1:** Patient characteristics.

	Case 1	Case 2	Case 3	Case 4
pTN status	pT2cN0	pT3aNx	pT3aN0	pT3bN0
Gleason score	3+4	4+3	4+4	4+5
Serum PSA level [ng/ml]	5.6	17.4	27.5	4.0
Non-surgical treatment	–	–	–	Preoperative ADT

Abbreviations: pTN, histopathologic tumor (T) and node (N) staging; PSA, prostate-specific antigen; ADT, androgen-deprivation therapy.

### Tissue Preparation

Xenografts and surgical biopsies from the prostatectomy specimens, fresh-frozen immediately following resection, were first evaluated for tumor cell content, after being sectioned using a cryostat microtome and stained with hematoxylin and eosin. Any normal tissue present in tumor samples was roughly removed by dissection guided by the stains. All tumor specimens used had tumor cell content higher than 80%. The specimens were subsequently cut in 10 µm sections, continuously kept frozen and immediately re-stored at −80°C. A volume of ∼3 mm^3^ was cut of all xenograft samples, the required number of sections to cut calculated after measuring the samples’ width and length. By identical procedures, ∼5 mm^3^ was sectioned of each prostatectomy biopsy (normal and tumor) specimen.

### Data Analysis of Prostate Carcinoma Xenografts’ Kinase Activity Profiles

Procedures for preparation of protein lysates and generation of kinase activity profiles are described in Text S1. Protein lysates from three biological replicates from each of the four experimental groups (ADT-naïve, AD, ADL, CR) were analyzed to generate the xenograft kinase activity profiles.

After visual check and background subtraction, endpoint signal intensities (the average signal intensity of the three last kinetic cycles) for each spot, i.e. for each kinase peptide substrate per array, were calculated by BioNavigator (PamGene International B.V.). A small number of negative signal intensities were managed by subtracting the 1% quantile of all data and setting remaining signal intensities less than 1 to 1. Subsequently, all signal intensities were log_2_-transformed, before the mean replicate signal intensity within each experiment was calculated for each peptide substrate. To correct for possible experiment-to-experiment variation, the mean of the four experimental groups’ signal intensities for each peptide substrate was subtracted from each group’s peptide substrate signal intensity. The microarray data are submitted to ArrayExpress (http://www.ebi.ac.uk/arrayexpress/); accession number E-MTAB-1572.

Statistical analysis was subsequently performed in PASW Statistics 18.0 (IBM, Somers, NY), with p<0.05 considered as statistically significant. ANOVA was applied to identify peptide substrates with significantly different phosphorylation levels between the experimental groups (ADT-naïve, AD, ADL, and CR). The Dunnett’s many-to-one (AD, ADL, and CR versus ADT-naïve) post-hoc test adjusted for multiple comparisons. The peptide substrates’ protein and gene names, including information about the cellular processes they are involved in, were retrieved using UniProt (http://uniprot.org; May 15, 2012) and HUGO Gene Nomenclature Committee (HGNC) (http://www.genenames.org; May 15, 2012) [16].

### Kinase Activity Profiling of Prostatectomy Biopsy Specimens

Three technical replicates (of both tumor and normal samples) were analyzed from each patient, on one Tyrosine PamChip®96 plate, at the PamGene International B.V. location in ‘s-Hertogenbosch, The Netherlands. Sample running was performed as previously described [17]. From the resulting data set, calculated after visual check and background subtraction, data were log_2_-transformed after setting a few data points with signal intensities less than 1 to 1.

### Hypoxic Exposure of Prostate Carcinoma Cells

The 22Rv1, PC3, and DU145 human prostate carcinoma cell lines were obtained from the American Type Culture Collection (ATCC). All cell lines were authenticated by short tandem repeat (STR) profiling based on 16 polymorphic markers in use by ATCC and paternity testing, and confirmed to be mycoplasma-free (MycoAlert, BioNordika, Oslo, Norway). All cell lines were maintained in RPMI 1640 medium (Sigma-Aldrich, Oslo, Norway) supplemented with 10% fetal calf serum, 1% L-Glutamine (Invitrogen, Oslo, Norway), and 1% penicillin/streptomycin (Invitrogen). The cells were routinely grown as a monolayer in 75 cm^2^ flasks at 37°C in 95% air/5% CO_2_, and subcultured twice weekly to maintain exponential growth.

To investigate changes in kinase activity induced by hypoxia, exponentially growing cells at ∼60% confluence, with fresh medium, were transferred to a hypoxia chamber for 24 hours (Invivo2 200; Ruskinn Technologies, Leeds, UK). Moderate hypoxia (1% O_2_) was achieved by 5% CO_2_ and residual N_2_. Severe hypoxia (anoxia) was obtained by 5% CO_2_ and a mixture of 10% H_2_ and 90% N_2_ in combination with a catalyst that catalyzed the conversion of H_2_ with any O_2_ left to H_2_O. All normoxia and hypoxia experiments were performed thrice in order to generate three biological replicates of all experimental conditions.

### Kinase Activity Profiling of Prostate Carcinoma Cells

Preparation of protein lysates is described in Text S1. Kinase activity profiling was done identically as for the xenografts, using 10 µg of total protein from all samples. Protein lysates from three biological replicates from each of the three experimental groups (normoxia, moderate hypoxia, severe hypoxia) were analyzed to generate the kinase activity profiles. In the post-hoc Dunnett’s test, the control condition was represented by the normoxic cells. An evaluation of the technical reproducibility of the arrays (interchip and intrachip correlations following analysis of the same cell culture lysate in all arrays) is presented in Text S2.

### Cobalt Chloride Treatment

To simulate hypoxic exposure and expression of the hypoxia-inducible factor-1α (HIF-1α) cells were exposed to 100 µM of the hypoxia-mimetic agent cobalt chloride (CoCl_2_) (Sigma Aldrich) for 4 hours before protein lysates were made. Treatment with CoCl_2_ induces HIF-1α expression by binding to the PAS domain, which blocks the HIF-1α pVHL binding, thereby giving HIF-1α stabilization [18].

### Western Blot Analysis

Protein and phosphoprotein expressions were measured by western immunoblots. The primary antibodies were anti-HIF-1α (BD Biosciences, Trondheim, Norway), anti-STAT5A (Sigma-Aldrich), and anti-phospho STAT5A/B (Y694/Y699) (Millipore, Temecula, CA). Mouse monoclonal anti-γ-tubulin (Sigma-Aldrich) was used as loading control. Secondary antibodies were peroxidase-conjugated goat anti-rabbit and donkey anti-mouse (Jackson ImmunoResearch Laboratories, West Grove, MA). Proteins were visualized by chemiluminescence using SuperSignal West Dura Extended Duration Substrate (Thermo Scientific, Rockford, IL) or LumiGLO Chemiluminescent Substrate (KPL, Gaithersburg, MD).

## Results

### Validation of the Preclinical Model

The CWR22 prostate carcinoma xenografts exhibited volume response patterns following castration similar to those seen in patients, and developed CR xenografts after 5.4±0.4 months (Figure 1A). When accounting for the about five times faster metabolism in mice, this corresponds to around 2–3 years in humans. The clinically relevant disease progression of the xenograft was further confirmed by serum PSA quantifications from the animals’ blood samples (Figure 1B). The reduction in PSA from ADT-naïve xenografts to AD and ADL xenografts were significant (p = 0.032 and p = 0.042, respectively). In CR xenografts, pre-ADT PSA levels were restored.

### Kinase Activity in Xenografts Following ADT

From analysis of the kinase activity profiles generated by the xenografts, phosphorylation of 35 of the kinase peptide substrates was found to be significantly affected (reduced or increased) after ADT by castration (Table 2). These substrates represented proteins involved in several cellular processes, such as angiogenesis (e.g. PDGFR, VEGFR, EPOR), survival, proliferation, and progression (e.g. MAPK, RAF, STAT), and adhesion, migration, and invasion (e.g. FER, FES, MET).

**Table 2:** Kinase peptide substrates with significantly affected (reduced or increased) phosphorylation levels by samples from androgen-deprived (AD), long-term AD (ADL), or castration-resistant (CR) prostate carcinoma xenografts, versus androgen-deprivation therapy (ADT)-naïve xenografts.

Kinase peptide substrate^a^	Peptide start position^b^	Peptide end position^b^	Site(s) of phosphorylation^c^	Gene name^d^	Common name^e^
**Angiogenesis** f					
EGFR	1103	1115	Y1110	EGFR	Epidermal growth factor receptor
EPHA4	921	933	Y928	EPHA4	Ephrin type A receptor 4
EPOR	361	373	Y368	EPOR	Erythropoietin receptor
PECA1	706	718	Y713	PECAM1	Platelet endothelial cell adhesion molecule
PGFRB	1002	1014	Y1009	PDGFRB	Platelet-derived growth factor receptor beta
PGFRB	768	780	Y771/775/778	PDGFRB	Platelet-derived growth factor receptor beta
PGFRB	771	783	Y771/775/778	PDGFRB	Platelet-derived growth factor receptor beta
P85A	600	612	Y607/S608	PIK3R1	Phosphatidylinositol 3-kinase regulatory subunit alpha
VGFR1	1326	1338	Y1327/1333	FLT1^g^	Vascular endothelial growth factor receptor 1
VGFR2	989	1001	Y996	KDR^g^	Vascular endothelial growth factor receptor 2
**Survival/proliferation/progression**					
41	654	666	Y660	EPB41	Erythrocyte membrane protein band 4.1
CDK2	8	20	Y15/19	CDK2	Cyclin-dependent kinase 2
ENOG	37	49	Y44	ENO2	Gamma enolase 2
FRK	380	392	Y387	FRK	Fyn-related kinase
MK01	180	192	Y187	MAPK1	Mitogen-activated protein kinase 1
MK10	216	228	Y223/228	MAPK10	Mitogen-activated protein kinase 10
NTRK2	696	708	Y702/706/707	NTRK2	Neutrotrophic tyrosine kinase receptor 2
PDPK1	2	14	Y9	PDPK1	3-phosphoinositide dependent protein kinase 1
PRGR	786	798	Y795	PGR	Progesterone receptor
PRRX2	202	214	Y208/214	PRRX2	Paired-related homeobox protein 2
RAF1	332	344	Y340/341	RAF1	Raf proto-oncogene serine/threonine protein kinase
RET	1022	1034	Y1029	RET	Ret proto-oncogene tyrosine protein kinase receptor
STAT1	694	706	Y701	STAT1	Signal transducer and activator of transcription 1
STA5A	687	699	Y694	STAT5A	Signal transducer and activator of transcription 5A
TYRO3	679	691	Y681/685/686	TYRO3	Tyro3 tyrosine protein kinase receptor
**Adhesion/migration/invasion**					
EFS	246	258	Y253	EFS	Embryonal Fyn-associated substrate
FER	707	719	Y714	FER	Fer proto-oncogene tyrosine protein kinase
FES	706	718	Y713	FES	Fes/Fps proto-oncogene tyrosine protein kinase
MET	1227	1239	Y1230/1234/1235	MET	Hepatocyte growth factor receptor
PAXI	111	123	Y118	PXN	Paxillin
PAXI	24	36	Y31/33	PXN	Paxillin
PLCG1	764	776	Y771/775	PLCG1	Phospholipase C gamma 1
SRC8	476	488	Y477/483	CTTN1	Src substrate protein p85 (cortactin)
SRC8	492	504	Y492/499/502	CTTN1	Src substrate protein p85 (cortactin)
**Other**					
CD79A	181	193	Y182/188	CD79A	B-cell antigen receptor complex-associated protein alpha-chain

a Kinase peptide substrate names on the Tyrosine Kinase PamChip® microarray.

b Start and end positions for the kinase peptide sequence within the protein.

c Site(s) of tyrosine phosphorylation within the protein.

d Corresponding gene names and ^e^common names, retrieved from UniProt or HUGO Gene Nomenclature Committee (HGNC).

f Kinase peptide substrates are grouped according to the main cellular process they are involved in.

g FLT1 and KDR are also known as VEGFR1 and VEGFR2.

**Figure 2:**
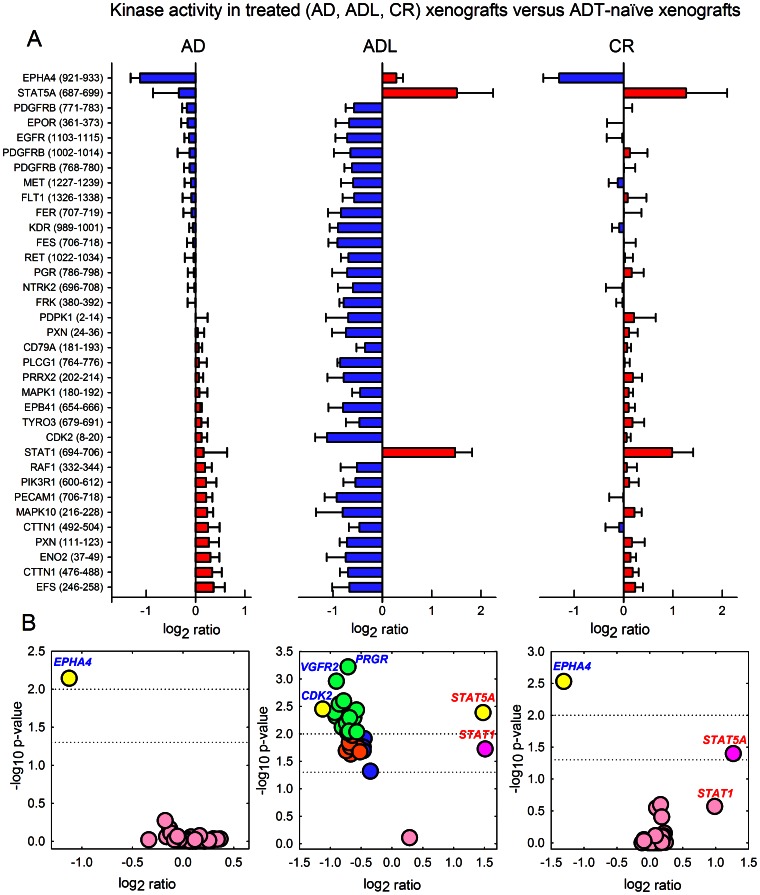
Kinase activity in xenografts following androgen-deprivation therapy (ADT). (A) Decreased (blue; negative log_2_ ratio) and increased (red; positive log_2_ ratio) phosphorylation levels of kinase peptide substrates by androgen-deprived (AD), long-term AD (ADL), and castration-resistant (CR) prostate carcinoma xenografts compared to ADT-naïve tumors (mean ratio and s.e.m. per substrate). Protein lysates from three biological replicates from each of the four experimental groups (ADT-naïve, AD, ADL, CR) were analyzed to generate the xenograft kinase activity profiles. Listed are the substrates’ corresponding gene names, with start and end positions for the peptide sequence within the protein in brackets. (B) Volcano plots (−log_10_ p-values versus ratios) visualizing kinase peptide substrates with most significant and/or highest log_2_ ratios between treated and ADT-naïve tumors’ phosphorylation levels. Color labeling: yellow: p<0.01 and ratio >1.0; green: p<0.01 and ratio = 0.5–1.0; dark pink: p = 0.01–0.05 and ratio >1.0; orange: p = 0.01–0.05 and ratio = 0.5–1.0; blue: p = 0.01–0.05 and ratio <0.5; light pink: p>0.05.

Compared to ADT-naïve xenografts, some substrate phosphorylation levels generated by AD xenografts were weakly decreased, while others were increased. In Figure 2A the mean log_2_ ratio and s.e.m. for all 35 significant substrates are shown for the three treatment groups versus ADT-naïve xenografts, with blue color indicating negative log_2_ ratio and red color indicating positive log_2_ ratio, respectively. In Figure 2B Volcano plots (-log_10_ p-values versus log_2_ ratios) are visualizing the kinase peptide substrates with the most significant and/or highest log_2_ ratios between treated and ADT-naïve tumors’ phosphorylation levels.

In accordance with the significant tumor growth inhibition following long-term ADT the level of substrate phosphorylation by ADL xenografts was strongly decreased for 32 of the 35 kinase peptide substrates, compared to the respective phosphorylation levels originating from exponentially growing ADT-naïve xenografts. Two of the three remaining kinase peptide substrates, representing the signal transducer and activator of transcription 1 and 5A (STAT1 and STAT5A), demonstrated significantly increased phosphorylation by ADL xenografts, with a log_2_ ratio versus ADT-naïve xenografts of 1.5±0.3 for STAT1 (p = 0.004) and 1.5±0.7 for STAT5A (p = 0.019). Phosphorylation by CR xenografts was for the majority of the substrates restored at the same levels as for ADT-naïve xenografts. However, also from the CR xenografts, the phosphorylation of the STAT1 and STAT5A substrates was high, for STAT5A being significant compared to ADT-naïve xenografts (p = 0.040; log_2_ ratio = 1.3±0.8).

### Kinase Activity in ADT-naïve Prostatectomy Samples

**Figure 3:**
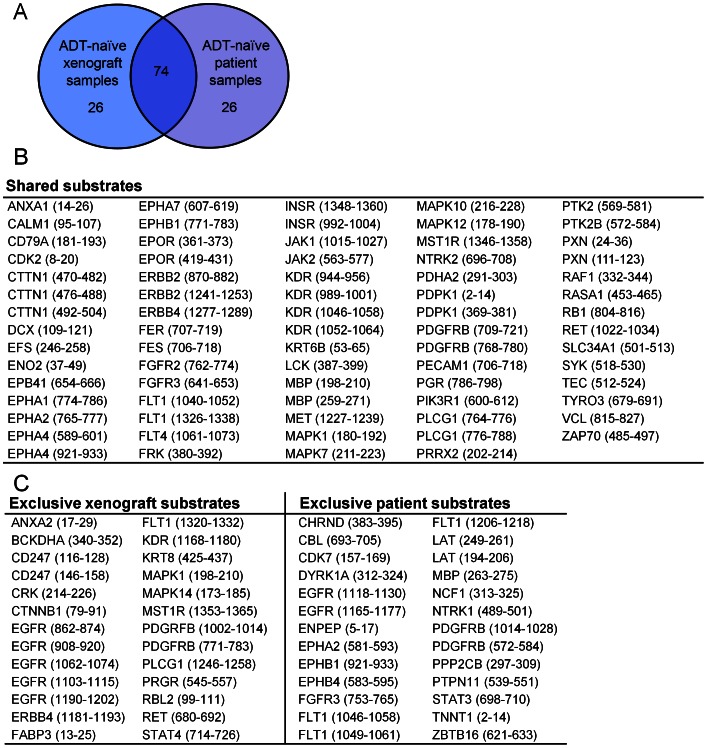
Kinase activity in androgen-deprivation therapy (ADT)-naïve xenografts versus ADT-naïve patient tumors. An ADT-naïve kinase activity signature was defined as the 100 kinase peptide substrates with highest phosphorylation levels. For prostate carcinoma xenografts, the signature was represented by the mean of all ADT-naïve substrate phosphorylation levels. For prostate cancer patients, a top 100 signature was produced by calculating the mean of the three ADT-naïve (case 1, 2, and 3; Table 1) tumor phosphorylation levels. (A) Seventy-four of the 100 substrates were shared by the xenograft and the patients’ tumor ADT-naïve signatures. (B) Phosphorylated kinase peptide substrates shared by ADT-naïve prostate carcinoma xenografts (n = 3) and ADT-naïve tumors from prostate cancer patients (mean of case 1, 2, and 3). Listed are the substrates’ corresponding gene names, with start and end positions for the peptide sequence within the protein in brackets. (C) The exclusively phosphorylated kinase peptide substrates (n = 26) in the xenograft signature (left) and in the patient tumor signature (right).

To assess clinical relevance, kinase activity profiles of prostatectomy samples from prostate cancer patients were generated. A kinase activity signature was retrieved from the 100 kinase peptide substrates with highest phosphorylation levels, for xenografts and patients’ tumor samples. For xenografts, the signature was represented by the mean of all ADT-naïve xenografts. For clinical samples, the signature was expressed by the mean of the three ADT-naïve tumor samples (cases 1, 2, and 3 in Table 1). The comparison of the two resulting signatures revealed 74 shared substrate phosphorylations (Figure 3A and B), whereas each of them comprised 26 exclusively phosphorylated substrates (Figure 3C). The 74% overlap indicates that the xenograft model used in the current study is a biologically relevant model of the clinical situation.

### STAT1 and STAT5A Substrate Phosphorylation by Prostatectomy Samples

**Figure 4:**
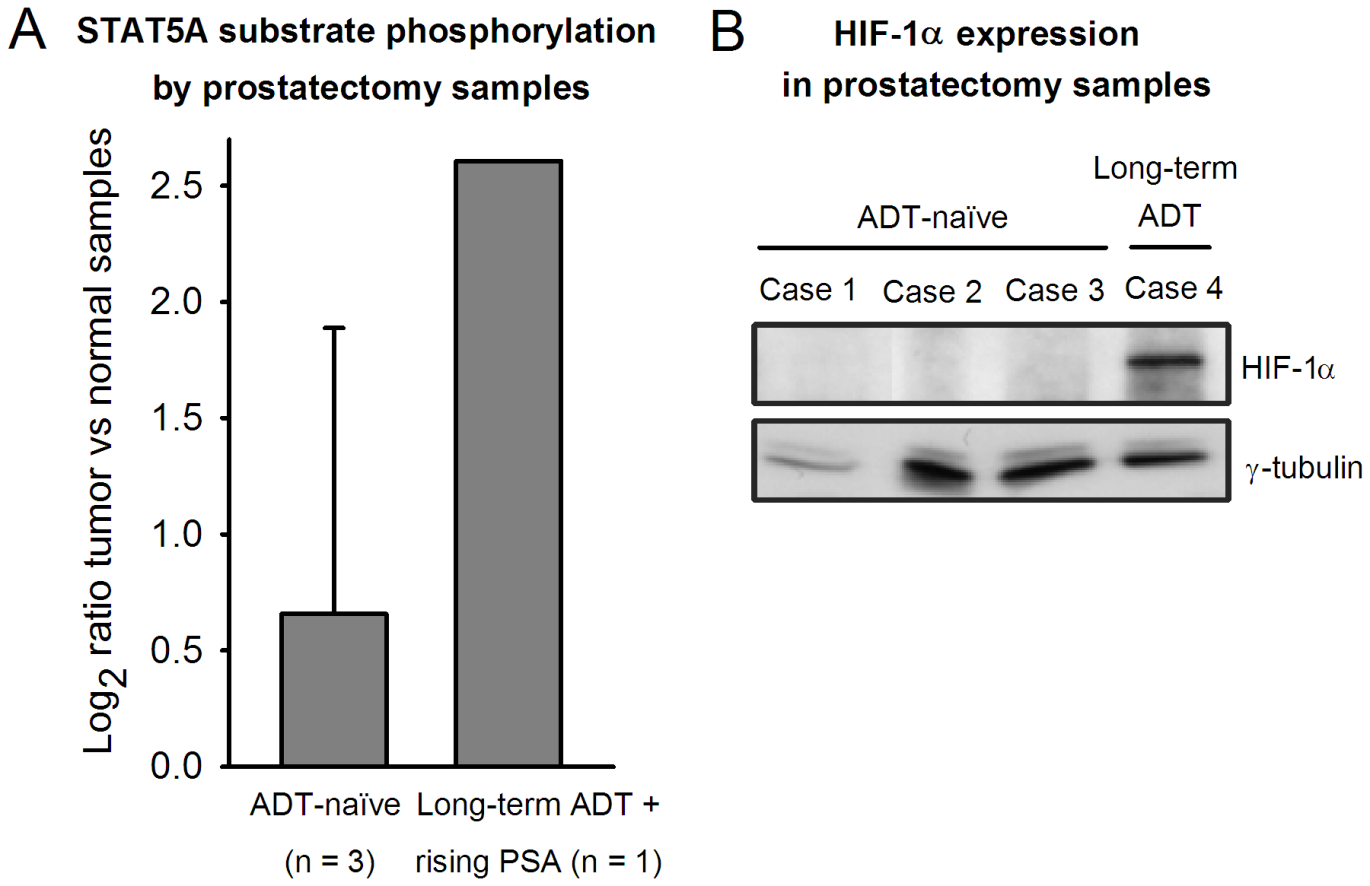
STAT5A substrate phosphorylation and HIF-1α expression in prostatectomy tumor samples. (A) For the androgen-deprivation therapy (ADT)-naïve prostate cancer patients, the log_2_ ratios between the substrate phosphorylation levels generated by the prostatectomy tumor sample versus the respective contralateral lobe normal tissue sample were calculated. For the patient that received long-term ADT before radical prostatectomy, the log_2_ ratio between the substrate phosphorylation levels from the tumor sample versus the mean substrate phosphorylation levels from the ADT-naïve patients’ normal tissue samples were calculated (since this patient’s normal tissue also was exposed to ADT). (B) Western immunoblot analysis of the prostatectomy tumor samples’ lysates with an antibody against hypoxia-inducible factor-1α (HIF-1α), the main protein known to be activated and stabilized under hypoxic conditions, revealing increased HIF-1α expression in the lysate from the early CR tumor (case 4). No HIF-1α expression was seen in lysates from ADT-naïve tumors.

After uncovering increased phosphorylation of the STAT1 and STAT5A substrates by ADL and CR xenografts compared to by ADT-naïve xenografts, the corresponding substrate phosphorylation levels from prostatectomy samples were investigated. To allow comparisons between different patients, the substrate phosphorylations by each of the three ADT-naïve patient’s tumor sample was normalized to the respective phosphorylations by the contralateral lobe normal tissue sample. One patient (case 4, Table 1) had received long-term ADT before early signs of CR disease were detected (rising serum PSA) and referral to radical prostatectomy. Since this patient’s normal tissue also was androgen-deprived, normalization of substrate phosphorylation generated by the tumor sample to that from the normal tissue sample was precluded. Thus, for this patient, substrate phosphorylations by the tumor sample were normalized to the mean of the normal tissue substrate phosphorylations by the three ADT-naïve patients. The STAT1 substrate phosphorylation was low and similar for all patients. However, whereas the STAT5A substrate phosphorylation by tumor versus normal samples in all of the ADT-naïve patients was low; log_2_ ratio = 0.7±1.2 (n = 3), it was substantially higher in the early CR patient; log_2_ ratio = 2.6 (n = 1) (Figure 4A).

### Hypoxia-induced Increase in STAT5A Substrate Phosphorylation by Prostate Carcinoma Cells

From the results achieved in xenografts and prostatectomies, combined with the recognition of the potential role of hypoxia in development of CR disease [5], it was hypothesized that hypoxia might be involved in the altered STAT5A kinase activity. To assess if the hypoxic state was present in the prostatectomy samples, Western immunoblot analysis of HIF-1α was performed on the prostatectomy tumor lysates. HIF-1α is the master regulator of oxygen homeostasis, known to be activated and stabilized under hypoxic conditions. Figure 4B shows that expression of HIF-1α was found in the lysate from the patient with early CR disease, as reflected by rising PSA following long-term ADT (case 4), whereas no HIF-1α expression was seen in the lysates from ADT-naïve tumors.

**Figure 5:**
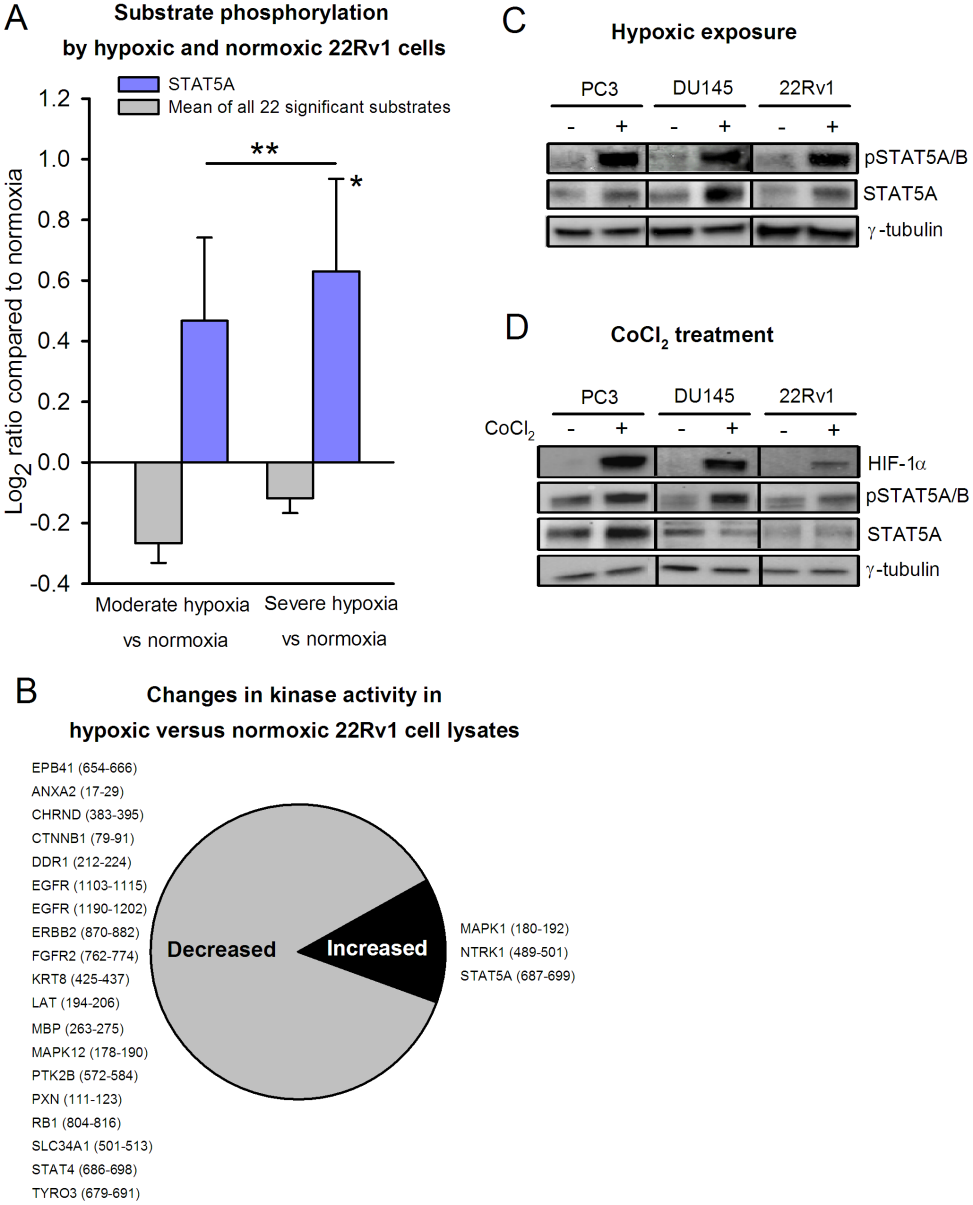
Hypoxia-induced increase in STAT5A substrate phosphorylation by prostate carcinoma cells. (A) 22Rv1 cells were exposed to moderate (1% O_2_) and severe (<0.02% O_2_) hypoxia for 24 hours in a hypoxia chamber. Protein lysates from three biological replicates from each of the three experimental groups (normoxia, moderate hypoxia, severe hypoxia) were analyzed to generate the kinase activity profiles. Kinase activity profiling of hypoxic and normoxic cell lysates detected increased STAT5A substrate phosphorylation with increasing hypoxia (blue bars), whereas the mean phosphorylation level for all 22 kinase peptide substrates with significant changes was low (grey bars). *The increase from normoxia to severe hypoxia was significant (p = 0.020). The difference in mean phosphorylation level at moderate versus severe hypoxia was not significant. (B) The proportion of decreased versus increased kinase activity in hypoxic versus normoxic 22Rv1 cell lysates. Listed are the gene names of the affected kinase peptide substrates, with start and end positions for the peptide sequence within the protein in brackets. (C) Western blot of anti-phospho-STAT5A/B and anti-STAT5A expression in normoxic (−) and severely hypoxic (<0.02% O_2_, 24 h) (+) cell lysates from PC3, DU145 and 22Rv1 cells. Anti-γ-tubulin served as loading control. (D) Western blot of anti-HIF-1α, anti-phospho-STAT5A/B and anti-STAT5A expression of lysates from PC3, DU145 and 22Rv1 cells with (+) or without (−) 4 hours of exposure to 100 µM of the hypoxia-mimetic agent cobalt chloride (CoCl_2_), a chemical inducer of HIF-1α. Anti-γ-tubulin served as loading control.

To further inspect this hypothesis, kinase activity profiles were generated from normoxic 22Rv1 prostate carcinoma cells, as well as from cells exposed to moderate (1% O_2_) and severe (<0.02% O_2_) hypoxia for 24 hours. The 22Rv1 cell line is androgen-responsive and originally derived from the relapsed CWR22 xenograft [19], expressing both the androgen receptor and PSA, thus representing a model of advanced prostate cancer. The phosphorylation levels of 22 kinase peptide substrates were significantly increased or decreased by hypoxic cell lysates (moderate or severe hypoxia) compared to by normoxic cell lysates. These included the STAT5A substrate, which presented an increasing phosphorylation level with increasing hypoxia (Figure 5A, blue bars), although the mean phosphorylation level for all the 22 significantly affected kinase peptide substrates was low (Figure 5A, grey bars). The difference in mean phosphorylation level at moderate versus severe hypoxia was not significant. For STAT5A, the log_2_ ratios compared to normoxic cells were 0.47±0.27 (p>0.05) for the moderately hypoxic cells and 0.63±0.31 (p = 0.020) for the severely hypoxic cells. A list of the 22 kinase peptide substrates significantly affected by hypoxia is provided in Figure 5B.

A Western immunoblot correspondingly confirmed an increased expression level of phospho-STAT5A/B (Y694/699) in severely hypoxic 22Rv1 cells, compared to normoxic 22Rv1 cells (Figure 5C). The expression of the total STAT5A protein was also found to increase under hypoxia, but to a lesser degree than the increase in phospho-STAT5A/B expression. To exclude that the finding of hypoxia-induced increased STAT5A activity was cell-line specific we analyzed two additional cell lines, the PC3 and DU145 prostate cancer cell lines, revealing similar results as in the 22Rv1 cell line (Figure 5C).

To elucidate whether STAT5A activation was directly induced by HIF-1α activation we treated the cells with CoCl_2_, a chemical inducer of HIF-1α. As seen in Figure 5D, CoCl_2_ induced increased expression of both HIF-1α and phospho-STAT5A/B in all cell lines. The STAT5A protein expression was increased in one cell line and unchanged in the two other cell lines. Since HIF-1α specifically induced an increase in STAT5 phosphorylation these results supports that hypoxia is the cause of increased STAT5 activity, through a HIF-1α-mediated mechanism.

## Discussion

The current study identified increased phosphorylation level of the STAT5A peptide substrate by ADL and CR prostate carcinoma xenografts, compared to by ADT-naïve xenografts. Similarly, in a limited population of prostate cancer patients studied, low phosphorylation levels of the STAT5A substrate by tumors from ADT-naïve patients’ were revealed, whereas the STAT5A phosphorylation level generated by the tumor from an early CR disease case was substantially higher. Thus, an association between high kinase activity mediated by STAT5A and development of CR disease was uncovered both in xenografts and patient tumor samples. Further, we detected higher phosphorylation levels of the STAT5A substrate by hypoxic prostate carcinoma cells than by the normoxic counterpart. Hypoxia-mediated activation of STAT5A signaling has to our knowledge not been reported in prostate cancer.

STAT5 belongs to the STAT gene family of transcription factors [19]. STAT5 comprises two highly homologous isoforms; the 94 kDa STAT5A and the 92 kDa STAT5B, being encoded by separate genes [20]. STAT5A and B (STAT5A/B) are acting both as cytoplasmic signaling proteins and as nuclear transcription factors. STAT5A/B is activated by phosphorylation of a tyrosine residue in the carboxy-terminal domain (Y694 for STAT5A and Y699 for STAT5B) upon stimulation of various mediators, often by a tyrosine kinase of the Janus kinase (JAK) protein family [20], [21]. After phosphorylation, STAT5A and STAT5B homodimerize or heterodimerize and translocate into the nucleus, where they bind to the STAT response element of target genes to regulate specific gene transcription [20], [22]. The STAT transcription factors are involved in regulation of several biological responses, including differentiation, proliferation, and migration [22].

Previous studies have identified STAT5A/B to be a critical survival factor in prostate cancer cells [23], and have further demonstrated increased STAT5A/B immunostaining in high-grade and CR clinical prostate tumors [24], [25], that active STAT5A/B in primary prostate cancer is predictive of early CR disease [26], and that transcriptional synergistic interaction between STAT5A/B and the androgen receptor inhibits apoptosis and promotes growth of prostate cancer cells *in vitro* [25]. Moreover, STAT5 is activated in a high percentage of clinical metastatic tumors, and activation of STAT5 in DU145 prostate carcinoma cells has been shown to cause substantially increased formation of experimental metastatic lung tumors [27]. Recently, these results were confirmed by the demonstration that STAT5 knockdown delayed development of CR disease in an *in vivo* preclinical model [28]. Our study verified the increased STAT5A-mediated kinase activity in CR prostate cancer xenografts. Further, we showed that phosphorylation of the STAT5A peptide was heightened already in ADL xenografts.

In liver cancer cells, hypoxia-induced activation of STAT5B was shown to regulate expression of insulin-like growth factors [29], which are known to be involved in cell survival and differentiation. In a study of microvascular endothelial cells, the JAK/STAT axis was shown to be activated by hypoxia and related to inhibition of a cellular pro-apoptotic pathway, and the results indicated that the JAK/STAT pathway plays an important role in endothelial cell survival during tissue hypoxia and angiogenesis [30]. Although the JAK/STAT signaling cascade often has been associated with immune function, recent studies have evidenced a role of JAK/STAT in mediating angiogenesis-related responses. For example, activation of STAT3 by the vascular endothelial growth factor (VEGF) was required for endothelial cell formation in a human *in vitro* model [31], and STAT5 was involved in interleukin-3-stimulated endothelial cell migration [32].

Hence, several studies propose a captivating link between the STAT proteins and survival of tumor endothelium during angiogenesis. Interestingly, in our study, several substrates representing kinases involved in angiogenesis were reactivated, i.e. presenting significantly higher level of substrate phosphorylation by CR xenografts compared to by ADL xenografts. These included PECAM1, several VEGFRs, PDGFRs, PIK3R1, and EPOR (Figure 2). Signaling commenced by VEGFRs is involved in the formation and development of endothelial cells, whereas PDGFR-signaling is engaged in stabilization of immature vessel walls by formation of pericytes [33], [34]. PDGFR, VEGFR and EPOR are also known to be activated by tumor hypoxia [4], [35]. Intriguingly, since hypoxia is suggested to promote development of CR disease in prostate cancer, STAT5; as was found to be significantly activated already in ADL xenografts in this study, may represent a key hypoxia-induced factor involved in subsequent reactivation of angiogenesis-related kinase signaling, which consequently stimulates renewed, CR tumor growth.

In summary, by using a novel peptide substrate microarray method, we were able to generate kinase activity profiles of preclinical and clinical samples from different stages in development of CR prostate cancer. From these profiles, previously published observations of high STAT5A activity in CR prostate cancer were confirmed. In addition, we also detected high STAT5A kinase activity already in regressing xenografts after long-term ADT. Since increased STAT5A kinase activity was generated by exposing prostate carcinoma cells to hypoxia, we propose that long-term ADT may induce tumor hypoxia and stimulate STAT5A kinase activity, which subsequently may lead to renewed, CR tumor growth. Hence, the study detected STAT5A as a candidate to be further investigated for its potential as marker of advanced prostate cancer and as possible therapeutic target protein.

## Supporting Information

Text S1 **Materials and methods.**
(DOC)

Text S2 **Evaluation of interchip and intrachip variation.**
(DOC)
