# Supplementary material for: Hypoxic Tumor Kinase Signaling Mediated by STAT5A in Development of Castration-Resistant Prostate Cancer
Source: PLoS One. 2013 May 10;8(5):e63723. doi: 10.1371/journal.pone.0063723 (PMC3651196; doi:10.1371/journal.pone.0063723)
Supplement: Text S1 — Materials and methods. (DOC) [file pone.0063723.s001.doc]

**Supporting Information Text S1: Materials and methods**

**Lysate preparation of xenografts**

Unless otherwise stated, all reagents were purchased from Sigma-Aldrich. Protein lysates were prepared by adding 100 µl of lysis buffer (M-PER (Mammalian Protein Extraction Reagent) containing Halt phosphatase inhibitor cocktail and EDTA-free Halt protease inhibitor cocktail (Fisher Scientific, Oslo, Norway) and mixed by pipetting and vortexing before 25 minutes of incubation on ice, followed by 15 minutes of centrifugation (15000 rpm, 4ºC). The resulting supernatant was filtered (Millex-GP, 0.22 µm; Millipore) before aliquoted into five vials, including one separate vial to protein concentration quantification, and immediately put on dry ice before being stored at -80ºC. Protein concentrations (of all samples simultaneously) were measured using a Micro BCA protein assay kit (Fisher Scientific).

**Lysate preparation of cells in culture**

To generate lysates of normoxic and hypoxic prostate carcinoma cells, the medium was removed before cells were washed with 10 ml ice-cold PBS twice. Then, after adding 4 ml ice-cold PBS, the cells were loosened by scraping and divided into four 1.5 ml vials. Cell pellets were obtained by centrifugation (10 minutes, 2500 rpm, 4ºC). The supernatant was removed and identical lysis buffer (150 µl/vial) as for the xenografts was added to each pellet. Following pipetting and vortexing, the cells were lysed for 15 minutes on ice. After centrifugation (15 minutes, 15000 rpm, 4ºC), resulting supernatants from individual samples were mixed in one vial before aliqouted and immediately frozen at -80ºC. Protein concentrations were measured using a Micro BCA protein assay kit (Fisher Scientific).

**Kinase activity profiling of prostate carcinoma xenografts**

Kinase activity profiling was performed at the PamStation® (PamGene International B.V., ‘s-Hertogenbosch, The Netherlands) facility at Akershus University Hospital, Lorenskog, Norway, simultaneously running three Tyrosine Kinase PamChip®4. Each array contains 144 kinase peptide substrates, representing about 100 different proteins. Protein lysates from three biological replicates from each of the four experimental groups (ADT-naïve, AD, ADL, CR) were used to generate the xenograft kinase activity profiles. Each PamChip® has four arrays and three PamChips® can be analyzed simultaneously on the PamStation®. Hence, lysates from one biological replicate from each of the four experimental groups (ADT-naïve, AD, ADL, and CR) were loaded in each of the four arrays on the first PamChip®. By correspondingly loading the same lysates on the second and third chip we could analyze three technical replicates of each biological sample simultaneously. The experiments were repeated to analyze all three biological replicates from the four experimental groups.

Prior to loading the sample reaction mixture, all 12 arrays (three PamChips® with four arrays each) were blocked with 25 µl of 2 % bovine serum albumin (BSA) solution each, and washed thrice with 1x protein kinase (PK) reaction buffer (diluted from 10x concentration in Ambion ultrapure water (Applied Biosystems, Oslo, Norway)). The sample reaction mixture consisted of 15 µg of total protein from each sample’s lysate, 4 µl of 10x PK buffer, 0.4 µl of 100x BSA solution, 0.4 µl of 1 mM dithiothreitol (DTT), 0.3 µl of monoclonal fluorescein isothiocyanate (FITC)-conjugated antibody PY20 (1 µg/µl in Tris buffer; PamGene International B.V., ‘s-Hertogenbosch, The Netherlands), 4 µl of 4 mM ATP, and ultrapure water adjusted to a total volume of 40 µl per array. Following loading of the sample reaction mixture, a manual kinetic protocol of 60 cycles of 60 seconds each was commenced, where the mixtures were pumped up and down through the porous arrays, enabling kinetic phosphorylations of the kinase peptide substrates by the samples to be recorded. For every fifth cycle, a charge-coupled device camera imaged the FITC fluorescence using a 50 ms exposure time. The fluorescence signal intensity per spot corresponds to the phosphorylation level of each kinase peptide substrate. During imaging, a wedge separates the unbound solution from the array, allowing imaging of bound solution only, i.e. of the phosphorylated kinase peptide substrates. After completing all the cycles, the arrays were washed twice with 1x PK buffer before end-level images with five different exposure times (10, 20, 50, 100, and 200 ms) were recorded.
