# Supplementary material for: Hypoxic Tumor Kinase Signaling Mediated by STAT5A in Development of Castration-Resistant Prostate Cancer
Source: PLoS One. 2013 May 10;8(5):e63723. doi: 10.1371/journal.pone.0063723 (PMC3651196; doi:10.1371/journal.pone.0063723)
Supplement: Text S2 — Evaluation of interchip and intrachip variation. (DOC) [file pone.0063723.s002.doc]

**Supporting Information Text S2: Evaluation of interchip and intrachip variation**

The variation between arrays and chips was evaluated by loading equal amounts of lysate from 22Rv1 prostate carcinoma cells in all 12 arrays (see illustration figure below). Interchip (Table 1) and intrachip (Table 2) correlations between peptide phosphorylation levels for the four arrays (A1 to A4) on the three simultaneously run PamChips® (C1 to C3) were assessed by Pearson’s correlations (*r*) of resulting signal intensities at the end of the 60 minutes incubation period.


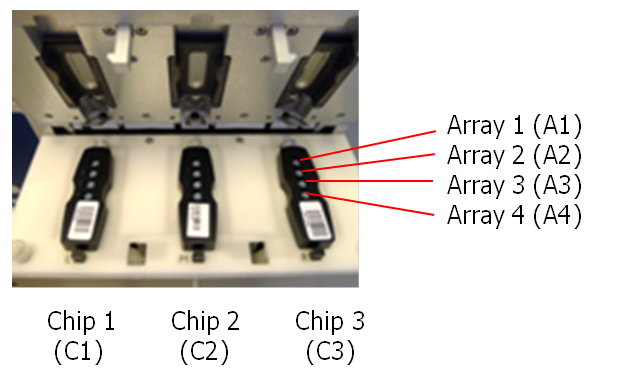


**Table 1.** Interchip correlations between 4 microarrays on 3 PamChips®

| C1 vs C2 | | C2 vs C3 | | C1 vs C3 | |
| --- | --- | --- | --- | --- | --- |
| Arrays | *r* | Arrays | *r* | Arrays | *r* |
| C1 A1 vs C2 A1 | 0.9967 | C2 A1 vs C3 A1 | 0.9983 | C1 A1 vs C3 A1 | 0.9965 |
| C1 A1 vs C2 A2 | 0.9952 | C2 A1 vs C3 A2 | 0.9977 | C1 A1 vs C3 A2 | 0.9950 |
| C1 A1 vs C2 A3 | 0.9940 | C2 A1 vs C3 A3 | 0.9983 | C1 A1 vs C3 A3 | 0.9960 |
| C1 A1 vs C2 A4 | 0.9968 | C2 A1 vs C3 A4 | 0.9963 | C1 A1 vs C3 A4 | 0.9957 |
| C1 A2 vs C2 A1 | 0.9932 | C2 A2 vs C3 A1 | 0.9958 | C1 A2 vs C3 A1 | 0.9928 |
| C1 A2 vs C2 A2 | 0.9931 | C2 A2 vs C3 A2 | 0.9974 | C1 A2 vs C3 A2 | 0.9908 |
| C1 A2 vs C2 A3 | 0.9918 | C2 A2 vs C3 A3 | 0.9968 | C1 A2 vs C3 A3 | 0.9918 |
| C1 A2 vs C2 A4 | 0.9944 | C2 A2 vs C3 A4 | 0.9979 | C1 A2 vs C3 A4 | 0.9926 |
| C1 A3 vs C2 A1 | 0.9936 | C2 A3 vs C3 A1 | 0.9965 | C1 A3 vs C3 A1 | 0.9933 |
| C1 A3 vs C2 A2 | 0.9953 | C2 A3 vs C3 A2 | 0.9981 | C1 A3 vs C3 A2 | 0.9927 |
| C1 A3 vs C2 A3 | 0.9935 | C2 A3 vs C3 A3 | 0.9968 | C1 A3 vs C3 A3 | 0.9932 |
| C1 A3 vs C2 A4 | 0.9952 | C2 A3 vs C3 A4 | 0.9977 | C1 A3 vs C3 A4 | 0.9944 |

**Table 2.** Intrachip correlations between 4 microarrays on 3 PamChips®

| Arrays | C1 | C2 | C3 |
| --- | --- | --- | --- |
| A1 vs A2 | 0.9971 | 0.9950 | 0.9982 |
| A1 vs A3 | 0.9962 | 0.9962 | 0.9985 |
| A1 vs A4 | 0.9972 | 0.9981 | 0.9971 |
| A2 vs A3 | 0.9964 | 0.9980 | 0.9990 |
| A2 vs A4 | 0.9973 | 0.9960 | 0.9984 |
| A3 vs A4 | 0.9975 | 0.9968 | 0.9980 |
